# MoMkk1 and MoAtg1 dichotomously regulating autophagy and pathogenicity through MoAtg9 phosphorylation in *Magnaporthe oryzae*

**DOI:** 10.1128/mbio.03344-23

**Published:** 2024-03-19

**Authors:** Yun Kong, Pusheng Guo, Jiayun Xu, Jiaxu Li, Miao Wu, Ziqi Zhang, Yifan Wang, Xinyu Liu, Leiyun Yang, Muxing Liu, Haifeng Zhang, Ping Wang, Zhengguang Zhang

**Affiliations:** 1 Department of Plant Pathology, College of Plant Protection, Nanjing Agricultural University, and Key Laboratory of Integrated Management of Crop Diseases and Pests, Ministry of Education, Nanjing, China; 2 The Key Laboratory of Plant Immunity, Nanjing Agricultural University, Nanjing, China; 3 Department of Microbiology, Immunology, and Parasitology, Louisiana State University Health Sciences Center, New Orleans, Louisiana, USA; The University of Texas Health Science Center at Houston, Houston, Texas, USA

**Keywords:** *Magnaporthe oryzae*, cell wall integrity, autophagy, protein phosphorylation, phospholipid

## Abstract

Autophagy is a central biodegradation pathway critical in eliminating intracellular cargo to maintain cellular homeostasis and improve stress resistance. At the same time, the key component of the mitogen-activated protein kinase cascade regulating cell wall integrity signaling MoMkk1 has an essential role in the autophagy of the rice blast fungus *Magnaporthe oryzae*. Still, the mechanism of how MoMkk1 regulates autophagy is unclear. Interestingly, we found that MoMkk1 regulates the autophagy protein MoAtg9 through phosphorylation. MoAtg9 is a transmembrane protein subjected to phosphorylation by autophagy-related protein kinase MoAtg1. Here, we provide evidence demonstrating that MoMkk1-dependent MoAtg9 phosphorylation is required for phospholipid translocation during isolation membrane stages of autophagosome formation, an autophagic process essential for the development and pathogenicity of the fungus. In contrast, MoAtg1-dependent phosphorylation of MoAtg9 negatively regulates this process, also impacting growth and pathogenicity. Our studies are the first to demonstrate that MoAtg9 is subject to MoMkk1 regulation through protein phosphorylation and that MoMkk1 and MoAtg1 dichotomously regulate autophagy to underlie the growth and pathogenicity of *M. oryzae*.

**IMPORTANCE:**

*Magnaporthe oryzae* utilizes multiple signaling pathways to promote colonization of host plants. MoMkk1, a cell wall integrity signaling kinase, plays an essential role in autophagy governed by a highly conserved autophagy kinase MoAtg1-mediated pathway. How MoMkk1 regulates autophagy in coordination with MoAtg1 remains elusive. Here, we provide evidence that MoMkk1 phosphorylates MoAtg9 to positively regulate phospholipid translocation during the isolation membrane or smaller membrane structures stage of autophagosome formation. This is in contrast to the negative regulation of MoAtg9 by MoAtg1 for the same process. Intriguingly, MoMkk1-mediated MoAtg9 phosphorylation enhances the fungal infection of rice, whereas MoAtg1-dependant MoAtg9 phosphorylation significantly attenuates it. Taken together, we revealed a novel mechanism of autophagy and virulence regulation by demonstrating the dichotomous functions of MoMkk1 and MoAtg1 in the regulation of fungal autophagy and pathogenicity.

## INTRODUCTION

Cell wall dynamics are important for cell growth, division, and adaptation to external changes (1, 2). To successfully colonize the host, *Magnaporthe oryzae* must respond and adapt to such external changes (3–7). Previous studies have shown that the cell wall in *M. oryzae* is constantly remodeled in a highly regulated and polarized manner by the cell wall integrity (CWI) signaling pathway (8, 9). CWI signaling is mediated by membrane-spanning sensors and a conserved mitogen-activated protein kinase (MAPK) signaling transduction cascade that includes kinases MoMck1, MoMkk1, and MoMps1 (3, 10, 11). MAPK signaling regulates the nuclear localization and activation of transcription factors, such as MoSwi6, MoSwi4, and MoMig1, under cell wall stress conditions (12). However, constitutively activated CWI signaling disrupts the balance between growth and stress response. Previous studies also showed that phosphatases MoPtc1 and MoPtc2 dephosphorylate MoMkk1 to switch off CWI signaling (13). Moreover, studies indicated that the MAPK cascade plays a role in CWI signaling and autophagy, which is regulated by MAPK pathways in response to external stimuli or environmental conditions (14). In *M. oryzae*, a previous study has shown that MAPK signaling is pivotal in regulating non-selective autophagy (15).

Autophagy is an evolutionarily conserved intracellular degradation process that plays a crucial role in maintaining internal homeostasis and providing nutrients through the delivery of proteins and membranes to lysosomes/vacuoles (16–18). In plant pathogenic fungi, autophagy is indispensable for survival and pathogenicity (19–21). The initiation and activation of the autophagy complex, consisting of autophagy-related (ATG) proteins Atg1, Atg13, and Atg17, are crucial for the autophagy process. These proteins localize to the phagophore assembly sites (PASs), where other autophagic proteins are recruited and assembled (22, 23). Within the PAS, vesicles are tethered and fused to form the cup-shaped phagophore, which then undergoes further extension and enclosure to form the central organelle autophagosome (24).

Autophagosomes are double-membrane structures formed by the phagophores sequestering cytoplasmic elements (25). Their formation involves a highly regulated and continuous membrane fusion process, including the generation of autophagosomal membrane precursors and phagophore elongation (24). Atg9, the conserved and only transmembrane autophagy-related protein, plays an essential role in autophagosome formation (24, 26, 27). In the budding yeast *Saccharomyces cerevisiae*, Atg9 has a multiple punctate location ubiquitously at the PAS, Golgi apparatus, mitochondria, and endosomes. It undergoes a quick turnover between these sites to increase autophagosome numbers (28). Atg9 vesicles from the Golgi apparatus provide the initial membrane source for autophagy at the early step of autophagosome formation (29–31). In addition, Atg9 vesicles form seeds that establish membrane contact sites to initiate lipid transfer from donor compartments, such as the endoplasmic reticulum (ER) (32, 33). By recruiting Atg2 and Atg18, the Atg9-Atg2-Atg18 complex colocalizes at the expanding edge of the isolation membrane (IM) (24, 34). At the same time. Atg2 physically tethers to the ER to transfer newly synthesized phospholipids to the cytoplasmic inner membrane leaflet (35–38). Among the complex subunits, Atg9 functions on the translocation of superfluous phospholipids from the cytoplasmic leaflet to the luminal leaflet using its lipid scramblase activity, thereby regulating autophagosome formation (39–41).

A previous study showed that autophagy is completely blocked in the *Moatg9* knockout mutant of *M. oryzae* (42), and MoAtg9 interacts with MoMkk1 (15). However, the role of MoAtg9 in autophagy and the regulatory mechanism of MoAtg9-MoMkk1 interaction in *M. oryzae* remain unknown. In this study, we found that MoMkk1 phosphorylates MoAtg9 to mediate autophagosomal membrane expansion during autophagy in contrast to MoAtg1-dependent MoAtg9 phosphorylation that suppresses the same process.

## RESULTS

### MoMkk1 interacts with and phosphorylates MoAtg9

**Fig 1:**
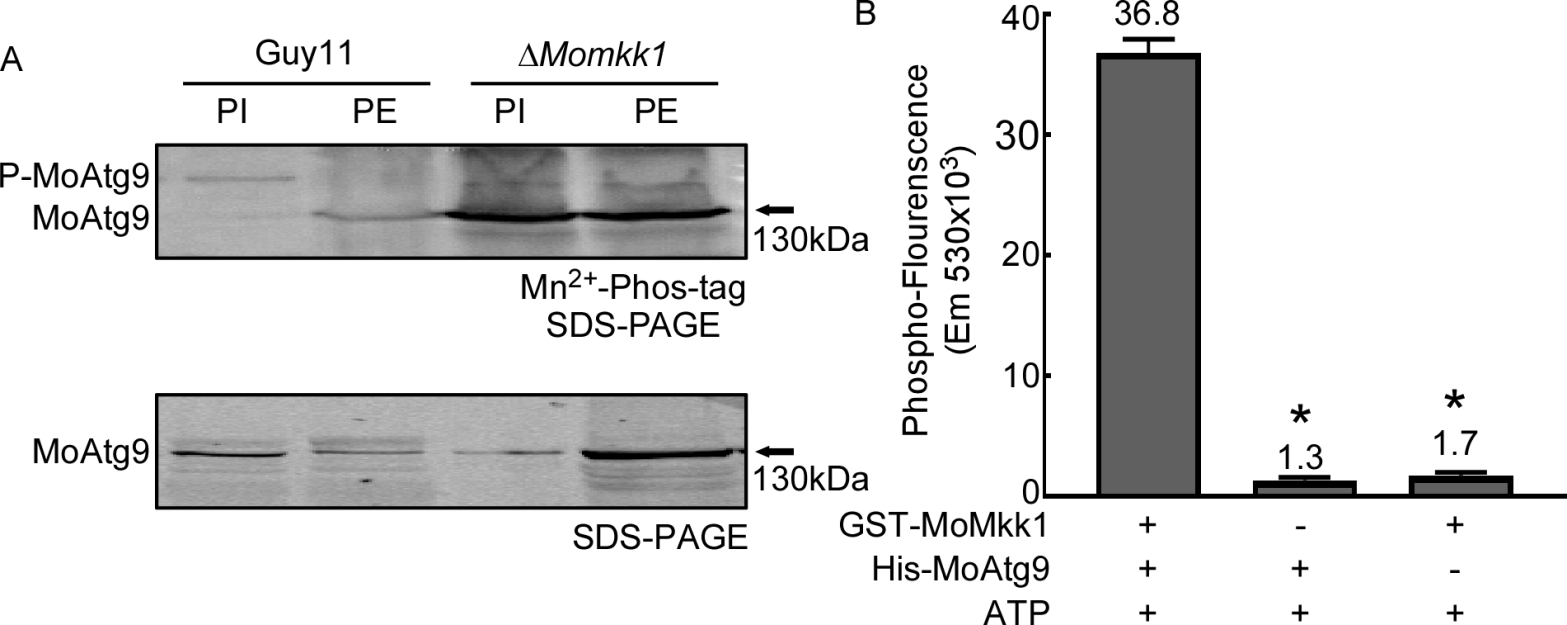
MoAtg9 is phosphorylated by MoMkk1. (**A**) *In vivo* phosphorylation analyses of MoAtg9-GFP proteins treated with phosphatase inhibitor (PI), phosphatase (PE), and detected by the anti-GFP antibody. (**B**) *In vitro* phosphorylation analysis by the fluorescence detection in tube (FDIT) method. Purified proteins of GST-MoMkk1 and His-MoAtg9 were used in protein kinase reactions in the presence of 50 µM ATP and then dyed with a Pro-Q Diamond Phosphorylation Gel Stain. The fluorescence signal at 590 nm (excited at 530 nm) was measured in a Cytation3 microplate reader. Error bars represent SD, and asterisks denote statistical significance (*P* < 0.01).

MoMkk1 is an important kinase in the CWI pathway that also plays a key role in mediating autophagy (15), which regulates pathogenicity through a series of ATG proteins in *M. oryzae* (43). We hypothesized that MoMkk1 functions in autophagy through interacting with ATG proteins. To test this hypothesis, we examined interactions between MoMkk1 and ATG proteins via yeast-two-hybrid (Y2H) and identified MoAtg9 as a MoMkk1-interacting protein (Fig. S1). MoAtg6, one of the ATG proteins in *M. oryzae*, was used as the negative control that had no interaction with MoMkk1 (Fig. S1). Since MoMkk1 is a protein kinase, we tested whether MoMkk1 phosphorylates MoAtg9. We transformed a MoAtg9-GFP construct into wild-type Guy11 and Δ*Momkk1* mutant strains and purified the MoAtg9-GFP fusion protein with anti-GFP beads. Phosphorylation analysis using Mn^2+^-Phos-tag SDS-PAGE and phosphatase inhibitors showed that more phosphorylated-MoAtg9 (P-MoAtg9) was present in Guy11 than the Δ*Momkk1* mutant strain (Fig. 1A), suggesting that phosphorylation of MoAtg9 is largely dependent on MoMkk1. This was confirmed by an *in vitro* phosphorylation assay using a protein gel-staining fluorescence dye (44). Co-incubation of purified GST-MoMkk1 and His-MoAtg9 proteins generated significantly higher phospho-fluorescence than the control (Figure 1B). These results demonstrated that MoMkk1, indeed, phosphorylates MoAtg9.

### MoMkk1-dependent MoAtg9 phosphorylation is essential for the development and pathogenicity of *M. oryzae*

**Fig 2:**
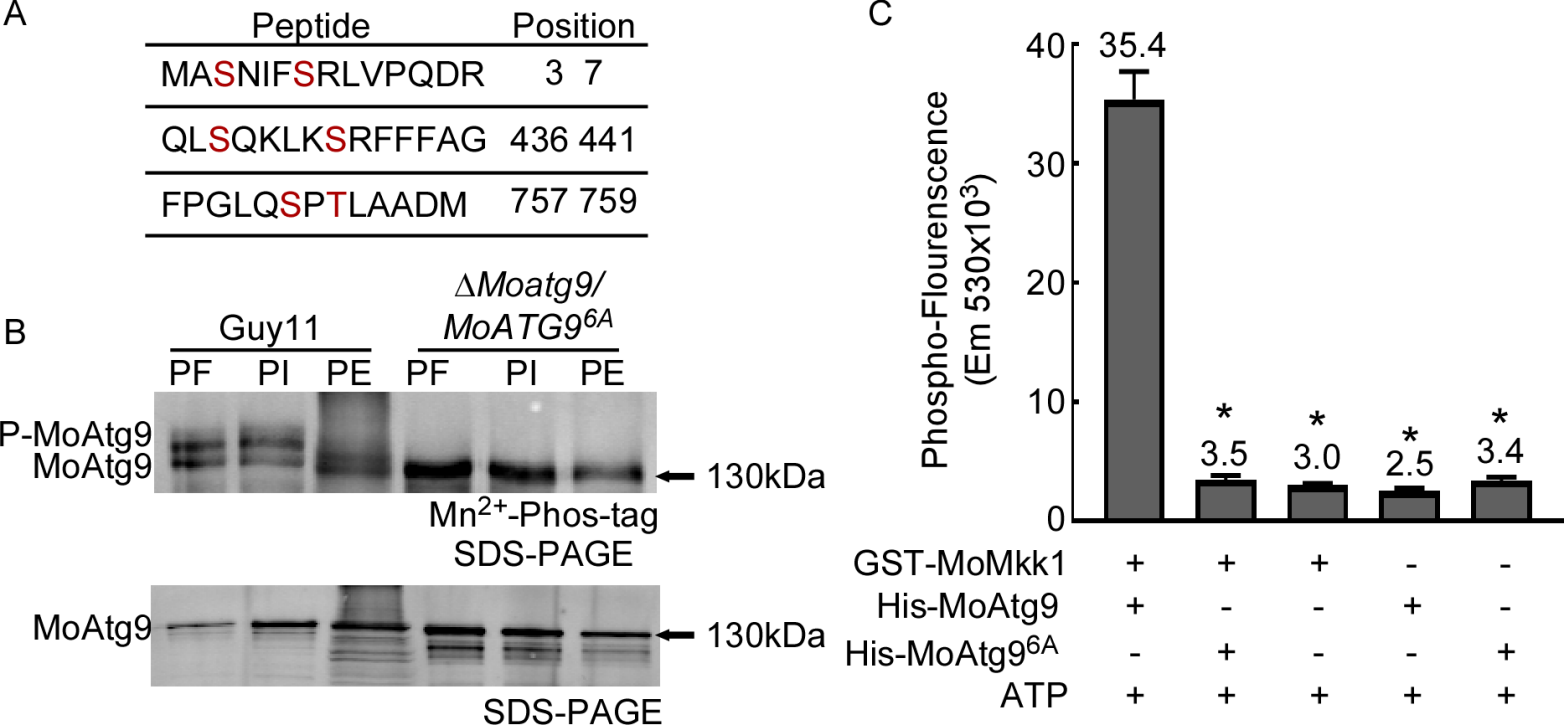
Verification of specific phosphorylation sites on MoMkk1. (**A**) Prediction of MoAtg9 phosphorylation sites from Guy11 and the Δ*Momkk1* mutant, indicated by red letters, were identified by LC-MS/MS analysis. (**B**) *In vivo* phosphorylation analysis of MoAtg9 from Guy11 and the Δ*Moatg9*/*MoATG9^6A^* mutant in the presence of PI and PE. Proteins were extracted in the presence of PMSF (PF) to prevent the degradation. (**C**) *In vitro* phosphorylation analysis using the FDIT method. GST-MoMkk1, His-MoAtg9, and constitutively unphosphorylated His-MoAtg9^6A^ fusion proteins were obtained. Error bars represent SD, and asterisks denote statistical significance (*P* < 0.01).

We further examined MoAtg9 phosphorylation site(s) by mass spectrometry (MS) analysis and identified 3S, 7S, 436S, 441S, 757S, and 759T as the putative phosphorylation sites (Fig. 2A). To validate this finding, a constitutively unphosphorylated MoAtg9^S3A, S7A, S436A, S441A, S757A, T759A^-GFP (hereafter MoAtg9^6A^-GFP) fusion construct was transformed into the Δ*Moatg9* mutant strain, and phosphorylation analysis showed that MoAtg9 is no longer phosphorylated (Fig. 2B). Additionally, co-incubation of GST-MoMkk1 with His-MoAtg9^6A^ exhibited significantly less phospho-fluorescence than His-MoAtg9 (Figure 2C). These results collectively suggested that the identified putative phosphorylation sites are important for the phosphorylation of MoAtg9 by MoMkk1.

Loss of MoAtg9 was found to cause defects in the development and pathogenicity of *M. oryzae* (44). To explore if MoMkk1-dependent MoAtg9 phosphorylation is involved in these processes, MoAtg9-GFP, MoAtg9^6A^-GFP, and MoAtg9^6D^-GFP (MoAtg9^S3D, S7D, S436D, S441D, S757D, T759D^-GFP, a constitutively phosphorylated form of MoAtg9), were transformed into the Δ*Moatg9* mutant. Constitutively phosphorylated MoAtg9 in Δ*Moatg9*/*MoATG9^6D^*, but not constitutively unphosphorylated MoAtg9 in Δ*Moatg9*/*MoATG9^6A^*, could rescue the defects of Δ*Moatg9* in growth (Fig. S2A) and conidiation (Fig. S2B). In addition, the Δ*Moatg9*/*MoATG9^6D^* strain caused more typical lesions in contrast to fewer and smaller lesions by Δ*Moatg9*/*MoATG9^6A^* and Δ*Moatg9* strains (Fig. S3A and B). These results demonstrated that MoMkk1-dependent MoAtg9 phosphorylation is essential for the development and pathogenicity of *M. oryzae*.

### MoMkk1-dependent MoAtg9 phosphorylation is required for the maintenance of autophagy

Correct assembly of Atg9 on the PAS is crucial in autophagy upon nitrogen starvation, which can be marked by RFP-MoApe1 fusion proteins. To test whether MoMkk1-dependent MoAtg9 phosphorylation affects MoAtg9 localization, we co-transformed RFP-MoApe1 with MoAtg9-GFP, MoAtg9^6A^-GFP, or MoAtg9^6D^-GFP into Δ*Moatg9* and observed subcellular localizations of MoApe1 and MoAtg9 under different conditions. Under nutrient-rich conditions, MoAtg9, MoAtg9^6A^, and MoAtg9^6D^ exhibited a 25% localization to PAS (Fig. S4). However, upon nitrogen starvation, the localization of MoAtg9 on PAS significantly escalated to approximately 59% and showed no significant differences in all of the strains (Fig. S4), indicating that phosphorylation did not affect the location of MoAtg9 on PAS.

**Fig 3:**
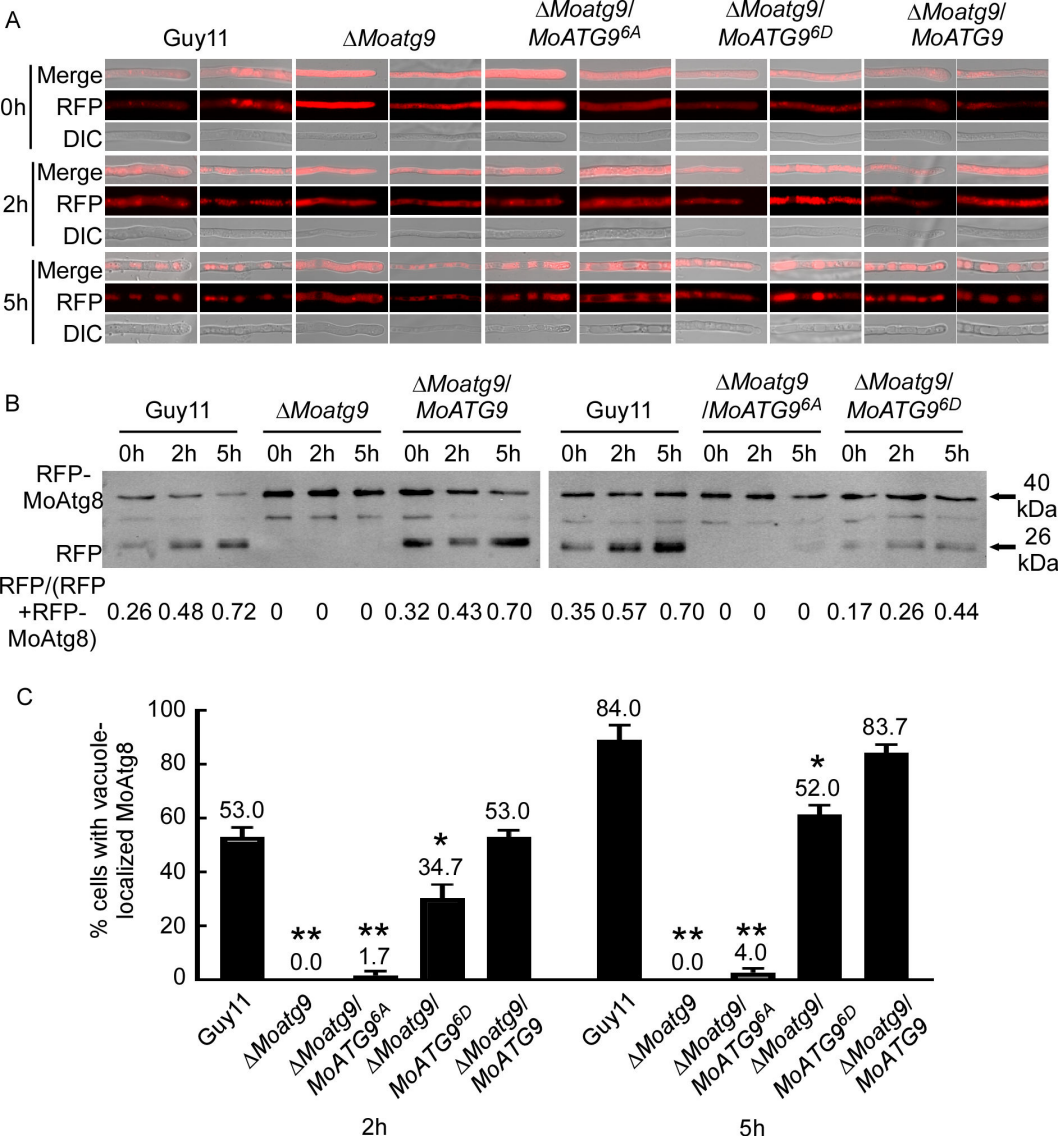
MoMkk1-dependent MoAtg9 phosphorylation could partially restore the defect of autophagy in Δ*Moatg9*. (**A**) Guy11, Δ*Moatg9*, Δ*Moatg9*/*MoATG9^6A^*, and Δ*Moatg9*/*MoATG9^6D^* strains transformed with RFP-MoAtg8 were cultured in MM-N (nitrogen starvation minimal medium) for 0, 2, and 5 h, and the autophagy intensity was observed by an Axio Observer A1 Zeiss inverted microscope. Scale bar, 10 µm. (**B**) The extent of autophagy was estimated by calculating the amount of free RFP compared with the total amount of intact RFP-MoAtg8 and free RFP (the numbers underneath the blot). (**C**) The autophagy intensity was assessed by means of the translocation of RFP-MoAtg8 into vacuoles (*n* = 100). Error bars represent SD, and asterisks represent statistical difference (*P* < 0.01).

**Fig 4:**
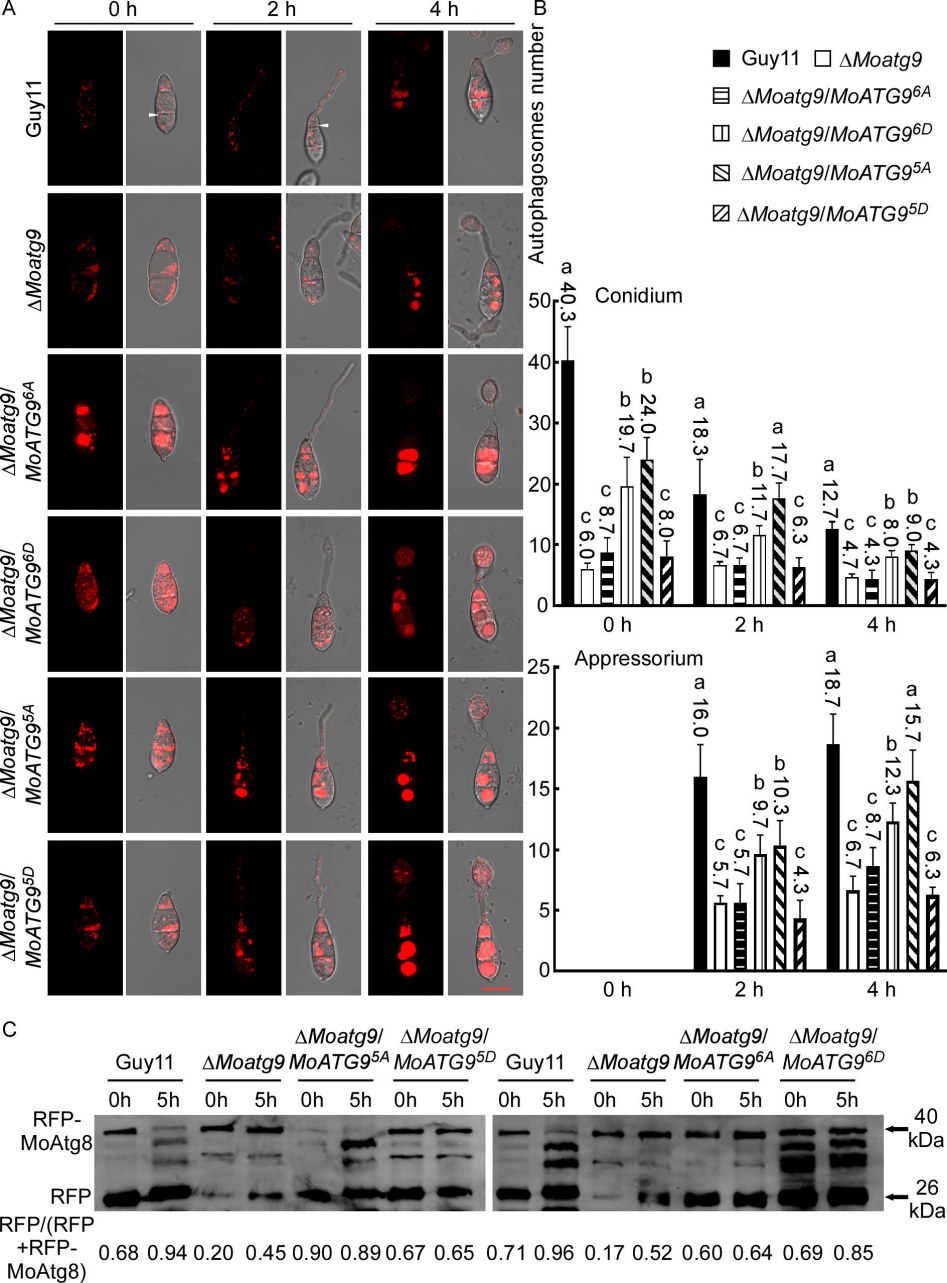
Subcellular localization of autophagosomes in conidia and appressoria. (**A**) Conidia from Guy11, Δ*Moatg9*, Δ*Moatg9/MoATG9^6A^*, Δ*Moatg9/MoATG9^6D^*, Δ*Moatg9/MoATG9^5A^*, and Δ*Moatg9/MoATG9^5D^* were inoculated onto hydrophobic interface for 2 and 4 h. The white arrow points to the autophagosomes. (**B**) The autophagy intensity was assessed by autophagosome numbers present in conidia and appressoria at 0, 2, and 4 h after germination. Error bars represent SD, and asterisks indicate statistical difference (*P* < 0.05). Scale bar, 10 µm. (**C**) The extent of autophagy was estimated by calculating the amount of free RFP compared with the total amount of intact RFP-MoAtg8 and free RFP (the numbers underneath the blot) for conidia or appressoria at 5 h after germination.

RFP-Atg8 was used to label autophagic structures at different stages, from the formation of autophagosomes to their fusion with lysosomes/vacuoles (45, 46). To further explore the role of MoMkk1-dependent MoAtg9 phosphorylation on autophagy, RFP-MoAtg8 introduced individually into Guy11, Δ*Moatg9*, Δ*Moatg9*/*MoATG9^6A^*, Δ*Moatg9*/*MoATG9^6D^*, and Δ*Moatg9*/*MoATG9* strains. Under rich nutrition conditions, RFP-MoAtg8 was distributed in the cytoplasm of all stains (Fig. 3A). However, after 2 h nitrogen starvation, RFP-MoAtg8 in Guy11, Δ*Moatg9*/*MoATG9^6D^*, and Δ*Moatg9*/*MoATG9* all formed apparent punctate structures in the cytoplasm, while its localization pattern remained unchanged in Δ*Moatg9* and Δ*Moatg9*/*MoATG9^6A^* (Fig. 3A). Further nitrogen deficiency treatment (5 h) showed that the accumulation of RFP-MoAtg8 in vacuoles of Guy11, Δ*Moatg9*/*MoATG9^6D^*, and Δ*Moatg9*/*MoATG9*, while it remained in the cytoplasm of Δ*Moatg9* and Δ*Moatg9*/*MoATG9^6A^* (Fig. 3A; Fig. S5). To confirm the essential role of MoAtg9 phosphorylation on autophagy, we examined the ratio of free RFP to the total amount of RFP-MoAtg8, which has been used as an autophagy level indicator, by Western blot analysis with the anti-GFP antibody. Δ*Moatg9* (0 h: 0, 2 h: 0, 5 h: 0) and Δ*Moatg9*/*MoATG9^6A^* (0 h: 0, 2 h: 0, 5 h: 0) had little effect on autophagy under starvation, while Δ*Moatg9*/*MoATG9^6D^* (0 h: 0.17, 2 h: 0.26, 5 h: 0.44) could partially compensate for autophagy defects (Fig. 3B). RFP-MoAtg8 was almost absent in vacuoles of Δ*Moatg9* (2 h: 0, 5 h: 0) and Δ*Moatg9*/*MoATG9^6A^* (2 h: 2%, 5 h: 3%) upon starvation induction (Figure 3C). However, Δ*Moatg9*/*MoATG9^6D^* could partially restore the autophagy defects, estimated to be 30% (2 h) and 61% (5 h) (Fig. 3C). Conidia adhere to the plant, causing severe blasts. Meanwhile, fluorescence observation also found that RFP-MoAtg8 increased remarkably in Δ*Moatg9*/*MoATG9^6D^* during germination (Fig. 4A and B). Similarly, the degradation of RFP-MoAtg8 was increased during germination by Western blot analysis with the anti-GFP antibody (Fig. 4C). The above findings demonstrated that MoMkk1-dependent MoAtg9 phosphorylation is required for restoring the autophagy defect of Δ*Moatg9*.

### MoAtg1 phosphorylates MoAtg9

**Fig 5:**
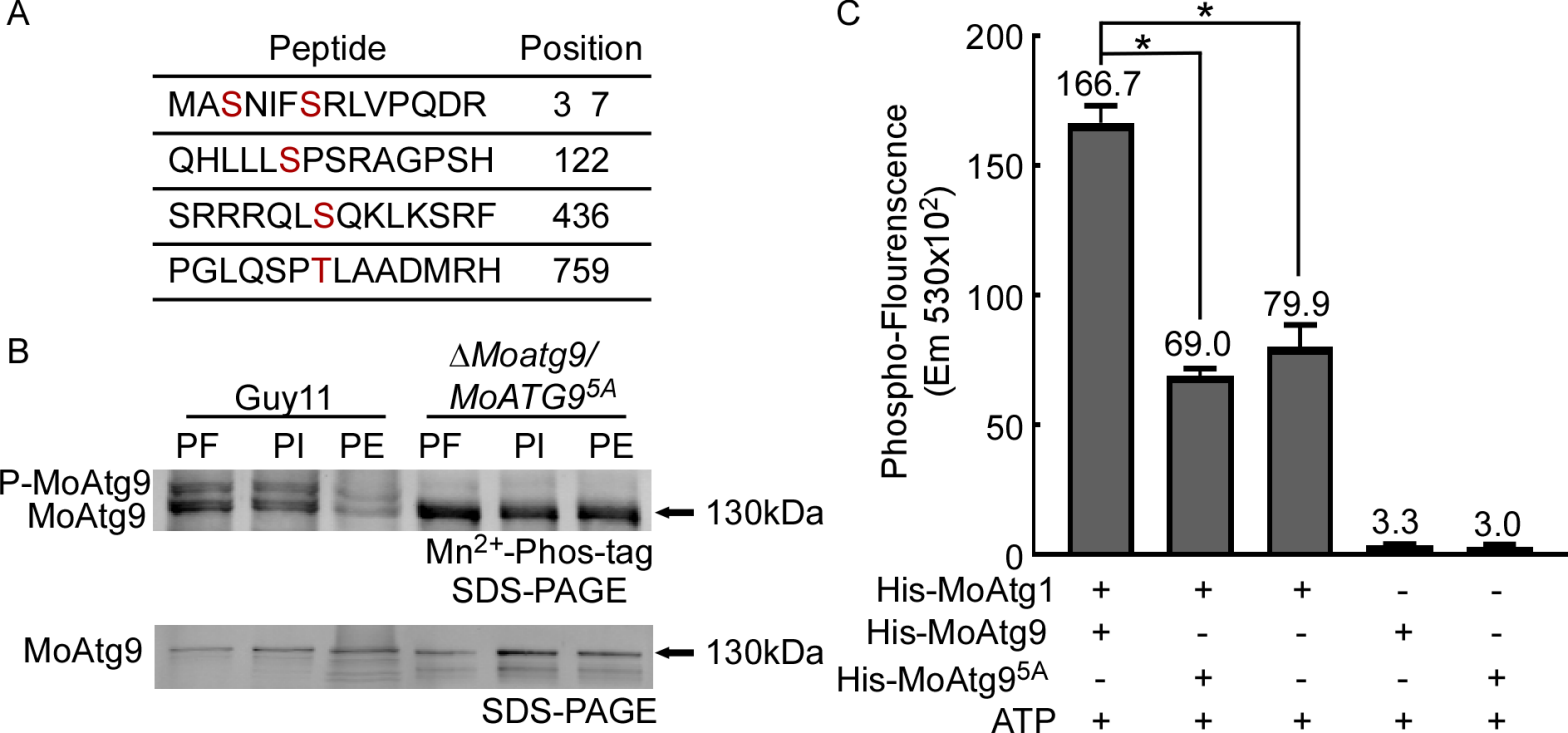
Verification of specific phosphorylation sites on MoAtg1. (**A**) Prediction of MoAtg9 phosphorylation sites, indicated by red letters, in Guy11 in comparison with the Δ*Moatg1* mutant expressing MoAtg9 was identified by LC-MS/MS analysis. (**B**) *In vivo* phosphorylation analysis of MoAtg9 in Guy11 and the Δ*Moatg1*/*MoATG9^5A^* mutant in the presence of PF, PI, and PE. (**C**) *In vitro* phosphorylation analysis using the FDIT method. His-MoAtg1, His-MoAtg9, and constitutively unphosphorylated His-MoAtg9^5A^ fusion proteins were obtained. Error bars represent SD, and asterisks denote statistical significance (*P* < 0.05).

Previous studies have shown that the serine-threonine kinase Atg1 is a key protein of the ATG protein complex, and Atg1-mediated phosphorylation of Atg9 is important in autophagy (47, 48). To investigate whether MoAtg1 could phosphorylate MoAtg9, we identified putative phosphorylation sites on MoAtg9 by MoAtg1 through MS analysis. Five putative phosphorylation sites (serine 3, 7, 122, 436, and threonine 759) were found (Fig. 5A). Four of these sites were the same as those for MoMkk1 phosphorylation. To test these phosphorylation sites, a constitutively unphosphorylated MoAtg9 ^S3A, S7A, S122A, S436A, T759A^-GFP (hereafter MoAtg9^5A^-GFP) fusion construct was expressed in the Δ*Moatg9* mutant. Phosphorylation of MoAtg9 (P-MoAtg9) was detected in Guy11 with phosphatase inhibitors but not in phosphatase-treated samples or the Δ*Moatg9*/*MoATG9^5A^* mutant (Fig. 5B). An *in vitro* phosphorylation showed that MoAtg1-dependent phosphorylation of MoAtg9^5A^ significantly decreased when compared to that of MoAtg9 (Fig. 5C). These results demonstrated that MoAtg1 could phosphorylate MoAtg9 in *M. oryzae*.

We then examined if MoAtg1-dependent phosphorylation of MoAtg9 affects the growth, asexual development, or pathogenicity of *M. oryzae*. MoAtg9-GFP, MoAtg9^5A^-GFP, and a constitutively phosphorylated MoAtg9 ^S3D, S7D, S122D, S436D, T759D^-GFP (hereafter MoAtg9^5D^-GFP) was individually introduced into Δ*Moatg9*. Surprisingly, we found that constitutively unphosphorylated MoAtg9^5A^ could partially rescue the defects of Δ*Moatg9* in growth (Fig. S2C), conidiation (Fig. S2D), and pathogenicity (Fig. S3A and C). But constitutively phosphorylated MoAtg9^5D^ caused fewer and restricted diseases compared to constitutively unphosphorylated MoAtg9^5A^ (Fig. S3A and C). These results indicated that MoAtg1-dependent phosphorylation of MoAtg9 negatively regulates the growth, development, and pathogenicity of *M. oryzae*.

### MoAtg1-dependent phosphorylation of MoAtg9 suppresses autophagy

As the core kinase of the autophagy pathway, Atg1 regulates different steps and factors in autophagy. Previous studies found that the anterograde trafficking of Atg9 to the PAS is adversely affected by Atg1 mutation (42, 49). To test if MoAtg9 phosphorylation by MoAtg1 affects the proper localization of MoAtg9 to PAS, RFP-MoApe1 was co-transformed individually with MoAtg9-GFP, MoAtg9^5A^-GFP, and MoAtg9^5D^-GFP into Δ*Moatg9*. Some of the MoAtg9-GFP, MoAtg9^5A^-GFP, and MoAtg9^5D^-GFP signals were present in the PAS following nitrogen starvation (Fig. S4B) with no significant differences found (Fig. S4C), indicating that MoAtg1-dependent MoAtg9 phosphorylation does not affect the location of MoAtg9 on PAS.

**Fig 6:**
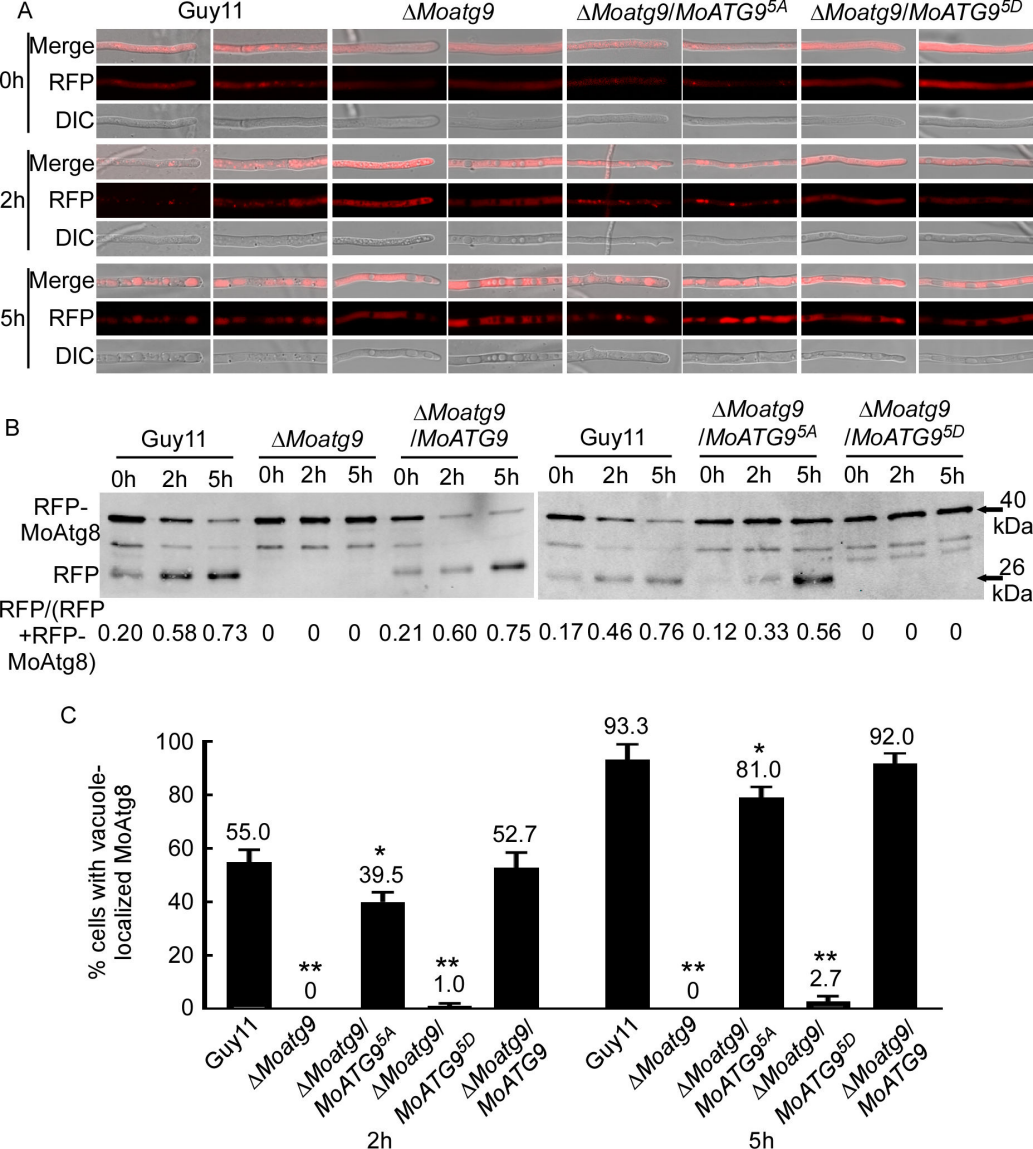
The non-phosphorylation mutation MoAtg9^5A^ could partially suppress the defect of autophagy in Δ*Moatg9*. (**A**) Guy11, Δ*Moatg9*, Δ*Moatg9*/*MoATG9^5A^*, and Δ*Moatg9*/*MoATG9^5D^* strains transformed with RFP-MoAtg8 were cultured in MM-N for 0, 2, and 5 h, and the autophagy intensity was observed using an Axio Observer A1 Zeiss inverted microscope. Scale bar, 10 µm. (**B**) The extent of autophagy was estimated by calculating the amount of free RFP compared with the total amount of intact RFP-MoAtg8 and free RFP (the numbers underneath the blot). (**C**) The autophagy intensity was assessed by means of translocation of RFP-MoAtg8 into vacuoles (*n* = 100). Error bars represent SD, and asterisks represent significant differences (*P* < 0.01).

To test whether MoAtg1-dependent MoAtg9 phosphorylation regulates autophagy, we observed the distribution pattern of RFP-MoAtg8 in hyphae of Δ*Moatg9*/*MoATG9^5A^* and Δ*Moatg9*/*MoATG9^5D^* strains under different conditions. Autophagic bodies marked by RFP-MoAtg8 were obviously aggregated after 2 h nutrient starvation in Guy11 and Δ*Moatg9*/*MoATG9^5A^* strains (Fig. 6A). Upon nutrient starvation for 5 h, diffused RFP signals could be observed inside the vacuoles of Guy11 and Δ*Moatg9*/*MoATG9^5A^* strains but not Δ*Moatg9*/*MoATG9^5D^* (Fig. 6A). The immunoblot assay confirmed that autophagy levels were remarkably increased in Δ*Moatg9*/*MoATG9^5A^* (2 h: 0.33, 5 h: 0.56) than Δ*Moatg9* (2 h: 0, 5 h: 0) and Δ*Moatg9*/*MoATG9^5D^* (2 h: 0, 5 h: 0) following nutrition starvation (Fig. 6B). In addition, vacuole-localized MoAtg8 was increased significantly in Δ*Moatg9*/*MoATG9^5A^* (2 h: 40%, 5 h: 79%), compared with Δ*Moatg9* (2 h: 0, 5 h: 0) and Δ*Moatg9/MoATG9^5D^* (2 h: 0, 5 h: 0) (Fig. 6C). Fluorescence observation also found that RFP-MoAtg8 increased remarkably in Δ*Moatg9*/*MoATG9^5A^* during germination (Fig. 4A and B). Similarly, the degradation of RFP-MoAtg8 was increased in Δ*Moatg9*/*MoATG9^5A^* during germination (Fig. 4C), indicating a higher autophagy level in Δ*Moatg9*/*MoATG9^5A^*. These results demonstrated that MoAtg1-dependent phosphorylation of MoAtg9 suppresses autophagy.

Considering the specific phosphorylation sites (441S, 757S, and 122S) phosphorylated by MoMkk1 and MoAtg1 play key roles in dichotomously regulation, we tested autophagy, development, and pathogenicity of MoAtg9^S122A^ and MoAtg9^S441D, S757D^. MoAtg9^S122A^ exhibited significant impairments in autophagy, development, and pathogenicity, while MoAtg9^S441D, S757D^ effectively suppressed these defects (Fig. S6). These findings collectively highlighted the pivotal roles of these two specific sites targeted by MoMkk1, whereas all five sites, including this one, demonstrated the significance of MoAtg9 phosphorylation by MoAtg1.

### MoAtg9 phosphorylation affects phospholipid translocation

**Fig 7:**
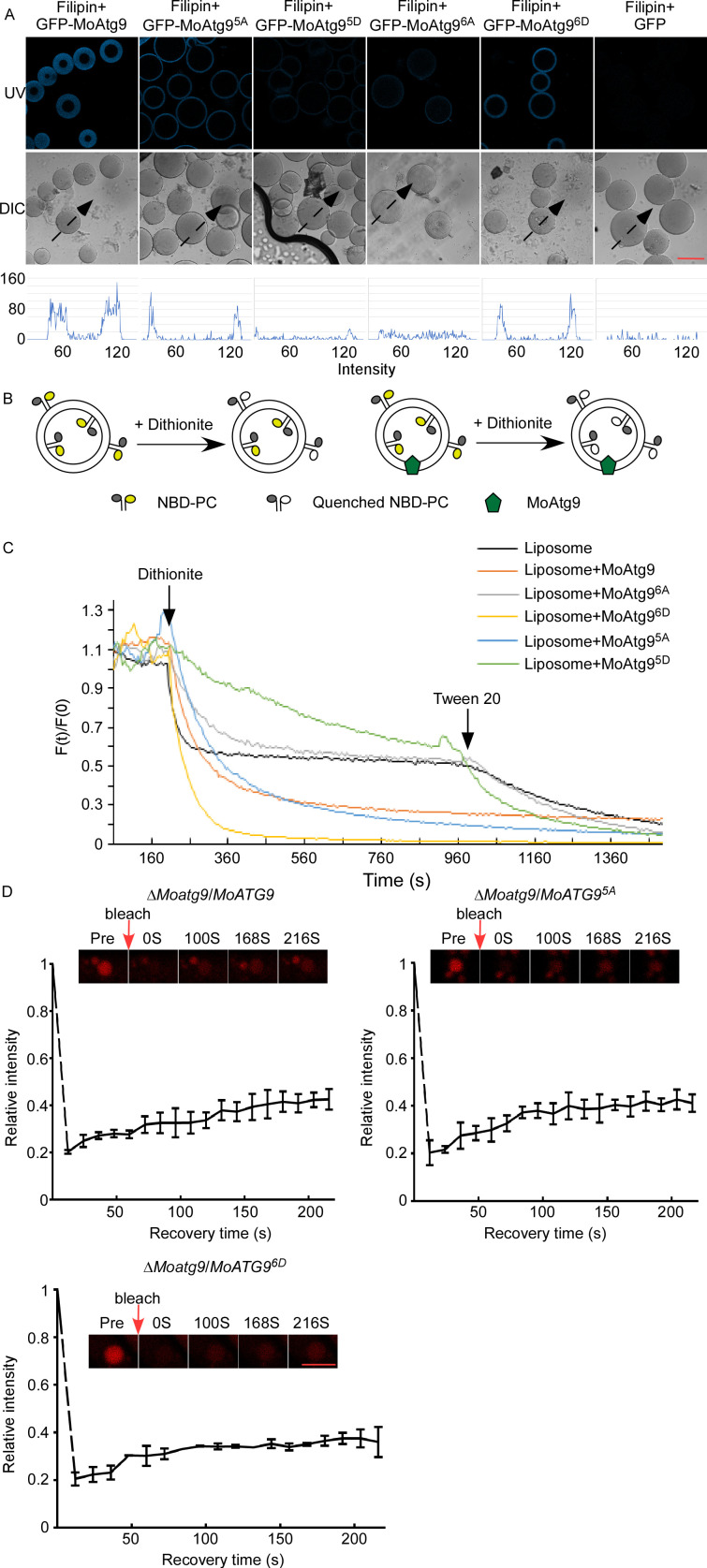
MoAtg9 phosphorylation affects phospholipid translocation. (**A**) The assay of protein binding to sterols. MoAtg9-GFP, MoAtg9^6A^-GFP, MoAtg9^6D^-GFP, MoAtg9^5A^-GFP, MoAtg9^5D^-GFP, and GFP were purified using GFP beads and fluorescence observed by a UV filter after staining with 50 µg/mL filipin for 4 h. Scale bar, 100 µm. (**B**) Schematic of the dithionite assay. (**C**) Results of the lipid scramblase assay. The fluorescence traces were obtained on protein-free liposomes and MoAtg9-containing liposomes with quenching. (**D**) FRAP assay of autophagosome marked RFP-MoAtg8. The scale bar represents 5 µm.

Previous studies showed that Atg9 is a lipid scramblase that translocates phospholipids between the outer and inner leaflets of liposomes in the process of the IM to autophagosome transition (33, 39). Filipin is a fluorescent probe that binds to sterols and forms a complex that could produce blue fluorescence (50–52). Based on this, we tested whether the binding of MoAtg9 to phospholipids is affected by MoMkk1/MoAtg1-dependent MoAtg9 phosphorylation during autophagosome formation. MoAtg9-GFP, MoAtg9^6A^-GFP, MoAtg9^6D^-GFP, MoAtg9^5A^-GFP, MoAtg9^5D^-GFP, and GFP proteins were purified using anti-GFP beads from Δ*Moatg9*/*MoATG9*, Δ*Moatg9*/*MoATG9^6A^*, Δ*Moatg9*/*MoATG9^6D^*, Δ*Moatg9*/*MoATG9^5A^*, Δ*Moatg9*/*MoATG9^5D^*, and Guy11/GFP, respectively, and beads were stained with filipin for UV fluorescence observation. We found that MoAtg9 exhibits a stronger fluorescence intensity than MoAtg9^6D^ and MoAtg9^5A^, while no fluorescence was observed for MoAtg9^6A^, MoAtg9^5D^, and GFP (Fig. 7A), supporting that MoMkk1 phosphorylated MoAtg9 could affect its binding ability and MoAtg1-dependent MoAtg9 phosphorylation is also important.

The characterization of MoAtg9 as a novel lipid scramblase was investigated using the dithionite assay (39, 53, 54). In this assay, liposomes containing NBD-PE [*N*-(7-nitrobenz-2-oxa-1,3-diazol-4-yl)-1,2-dihexadecanoyl-snglycero-3-phosphoethanolamine, triethylammonium salt], a lipid fluorescent probe, were utilized to irreversibly quench fluorescence within the outer leaflet of NBD-lipids upon treatment with dithionite (Fig. 7B left). MoAtg9-containing NBD-lipids are treated with dithionite, which quenches fluorescence within both leaflets (Fig. 7B right). Our observations demonstrated that liposomes with MoAtg9^5D^ and MoAtg9^6A^ exhibited approximately 50% less fluorescence post-dithionite treatment (Fig. 7C), indicating specific quenching of the outer leaflet. Meanwhile, dithionite treatment of MoAtg9-containing liposomes, MoAtg9^6D^-containing liposomes, and MoAtg9^5A^-containing liposomes quenched more than 50% of fluorescence (Fig. 7C). These data indicated that MoAtg9 is a lipid scramblase, and the function of MoAtg9 is regulated by phosphorylation of MoMkk1 and MoAtg1.

Based on the results showed in Figure 6A and 3A, RFP-MoAtg8 shows apparent fluorescence accumulation in Δ*Moatg9*/*MoATG9*, Δ*Moatg9*/*MoATG9^5A^*, and Δ*Moatg9*/*MoATG9^6D^* but not Δ*Moatg9*/*MoATG9^6A^* and Δ*Moatg9*/*MoATG9^5D^*. This observation indicated a pivotal role of MoAtg9 in phospholipid transport for autophagosome formation. In addition, FRAP (fluorescence recovery after photobleaching) was used to further verify that MoAtg9 phosphorylation affects the lipid transport. After FRAP, RFP-MoAtg8 fluorescence signals were recovered in Δ*Moatg9*/*MoATG9*, Δ*Moatg9*/*MoATG9^5A^*, and Δ*Moatg9*/*MoATG9^6D^* (Fig. 7D). This observation revealed the involvement of MoMkk1 or MoAtg1 phosphorylation of MoAtg9 in facilitating phospholipid translocation critical for autophagy in *M. oryzae*.

## DISCUSSION

**Fig 8:**
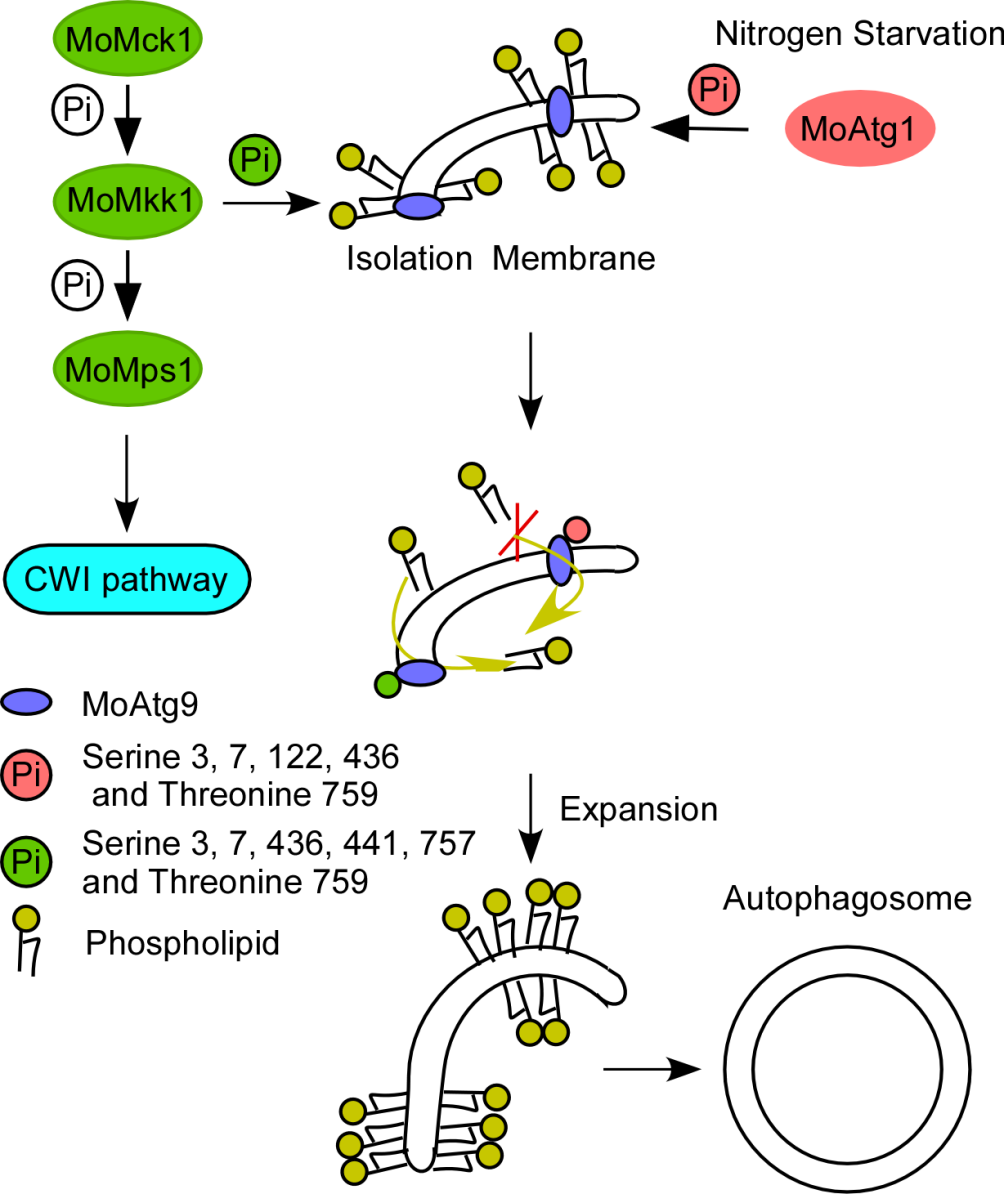
A model of *M. oryzae* utilizing MoMkk1/MoAtg1-dependent MoAtg9 phosphorylation to stimulate autophagosome formation in autophagy. MoAtg9 plays a key role in autophagosome formation during autophagy. When MoMkk1 is active, MoAtg1 is suppressed, and vice versa. MoMkk1 activation leads to dominant MoMkk1-dependent MoAtg9 phosphorylation that facilitates the transport of phospholipids and continuous inner membrane growth. In contrast, activated MoAtg1 leads to MoAtg1-dependent MoAtg9 phosphorylation that inhibits the accumulation of phospholipids in the inner membrane, thereby bending the isolation membrane and ensuring the formation of autophagosomes.

Autophagy is an important cellular process for the growth, development, and stress responses of *M. oryzae*. Under normal conditions, autophagy occurs at low intensity, but it is dramatically intensified in response to various stresses (55). It has been demonstrated that autophagy activates the CWI pathway under ER stress (15), but the mechanism of CWI signaling in autophagy is unknown. In this study, we found a core component of the CWI pathway, MoMkk1, phosphorylates a key autophagy-related protein MoAtg9 in *M. oryzae*. For a long time, canonical autophagy pathways have been found to be dependent on the ATG kinase Atg1. Our findings uncovered a novel mechanism by which MoMkk1 phosphorylates MoAtg9 to regulate the formation of autophagosomes with the expansion of IM during autophagy (Fig. 8). This is in conjunction with an opposing role by MoAtg1 that phosphorylates MoAtg9 to suppress the progress of IM expansion in autophagy (Fig. 8).

*M. oryzae* activates autophagy to counter host-imposed stress and facilitate infection (25, 56, 57). In yeast and mammalian cells, Atg1 is the conserved protein kinase that phosphorylates Atg9 to positively regulate autophagy (47, 58). Indeed, we found MoAtg9 could also be phosphorylated by MoAtg1 in *M. oryzae*. However, MoAtg1-dependent MoAtg9 phosphorylation negatively regulates growth, development, and pathogenicity in addition to autophagy in *M. oryzae* (Fig. S2 and S3; Figure 6).

A previous study in yeast has shown that an unknown kinase could also phosphorylate Atg9 (59). Interestingly, we found that MoMkk1 phosphorylates MoAtg9, and this phosphorylation positively regulates the development, pathogenicity, and autophagy of *M. oryzae* (Fig. S2 and S3; Fig. 3). Therefore, our study revealed a novel regulatory mechanism of MoAtg9 regulation that opened up the possibility of multiple pathway Atg9 regulation among various organisms. MoAtg9 phosphorylation by MoMkk1 and MoAtg1 dichotomously regulates autophagy and pathogenicity. RFP-MoAtg8 was distributed in hyphae without aggregation after 2 h nutrient starvation in Δ*Moatg9*, Δ*Moatg9/MoATG9^6A^*, and Δ*Moatg9/MoATG9^5D^* strains (Fig. 3 and 6). Atg9 is known to be required for the efficient recruitment of Atg8 to the site of autophagosome formation (47). The absence of MoAtg9 blocks autophagy, but MoAtg8 still aggregates and enters vacuoles for degradation during germination in *M. oryzae* (57). This suggests that MoAtg8 might be involved in processes other than non-selective autophagy during germination. Research has also shown that MoAtg8 is involved in glycogen autophagy of *M. oryzae* during appressorium development (3), and the glycogen degradation process is delayed but still occurs in the Δ*Moatg9* mutant (2), thus MoAtg8 exhibits different functions in hyphae and spores. Further examination revealed that defects in *∆Moatg9* were partially restored in MoAtg9^S441D, S757D^, similar to MoAtg9^6D^, but not MoAtg9^S122A^ (Fig. S6). These results highlighted that distinct and diverse biological consequences arise from specific phosphorylation combinations of the Atg9 protein (60).

On the other hand, the remaining four residues (S3, S7, S436, and S759) can be phosphorylated by both MoMkk1 and MoAtg1. However, when combined with S441 and S757, these residues play a positive role in autophagy (Fig. S6A). Notably, S122, phosphorylated by MoAtg1 only, plays a negative role in autophagy (Fig. S6A). The inability of S122A alone to rescue autophagy suggests that dephosphorylation of the other four residues is also necessary for inducing autophagy. When the S144D and S757D mutations constructs were transferred into the *Moatg9* mutant, the native MoMkk1 and MoAtg1 are, indeed, present and capable of phosphorylating the remaining four serine residues. Thus, during the process of MoAtg9 participating in autophagy and MoAtg9^6D^ inducing autophagy (Fig. 3), S122 is in a dephosphorylated status. Additionally, MoAtg9^5D^ suppresses autophagy (Fig. 6), indicating that S441 and S757 are in a dephosphorylated status. Conversely, MoAtg9^5A^ induces autophagy (Fig. 6), and MoAtg9^6A^ suppresses autophagy (Fig. 3), suggesting that S441 and S757 were in a phosphorylated status.

Atg9 phosphorylation plays a key role in autophagosome formation in autophagy (59). The PAS serves as the site for autophagosome generation (29, 61). In this study, the phosphorylation of MoAtg9 by MoMkk1 or MoAtg1 could still be co-located with the PAS marker MoApe1 under nitrogen starvation (Fig. S4), suggesting that phosphorylation of MoAtg9 does not affect MoAtg9 localization to PAS. We further demonstrated that MoMkk1-dependent MoAtg9 phosphorylation affects MoAtg9 lipid scramblase for IM expansion during autophagosome formation, which is antagonized by MoAtg1-dependent MoAtg9 phosphorylation (Fig. 7). In yeast, Atg1-dependent Atg9 phosphorylation is responsible for recruiting sufficient Atg18 for IM elongation (47). However, we found that Atg9 phosphorylation directly regulates IM expansion through its scramblase activity, not through recruiting other autophagy-related proteins. Atg9 forms a homotrimer with a very large pore for IM expansion (39, 54). We found that the conserved sites for homotrimer and pore formation are not phosphorylated by MoMkk1 or MoAtg1 (Fig. 2A and 5A). It is likely that MoAtg9 phosphorylation affects IM expansion without affecting the homotrimer structure or pore formation of MoAtg9.

Previous studies indicated that Atg9 functions as a crucial lipid transporter in orchestrating autophagosome formation, but the intricate regulatory mechanisms remain elusive (39, 40). Our studies demonstrated that MoMkk1 phosphorylates MoAtg9 to translocate phospholipids, resulting in IM expansion (Fig. 7). Previous research also showed that the temporal control of Atg9 phosphorylation is imperative for the autophagy processes (47). Continuous Atg9 activities potentially leading to adverse effects suggest a precise regulation of Atg9 scramblase activities, which is likely contingent upon membrane curvature dynamics (38). This regulation is dependent on Atg1, functioning as a membrane-tethering factor that exhibits selective lipid binding when membrane curvature is notably high (62). We reasoned that, once recruited to the membrane, MoAtg1 phosphorylates MoAtg9 to suppress phospholipid translocation. Consequently, MoAtg1-mediated phosphorylation of MoAtg9 emerges as a critical regulatory checkpoint, preserving the requisite membrane curvature essential for efficient autophagosome formation.

## MATERIALS AND METHODS

### Strains and cultural conditions

The *M. oryzae* Guy11 strain was used as wild type (WT) in this study. All strains were cultured on complete medium (CM) for 3–7 days in the dark at 28°C. For vegetative growth, 2 mm × 2 mm agar blocks were cut and placed onto fresh media, followed by incubation for 7 days in the dark at 28°C. Mycelia were harvested from the liquid CM media with or without additional treatment for DNA, RNA, and total protein extractions. For conidia production, strains were cultured on straw decoction and corn (SDC) agar media at 28°C for 7 days in the dark, followed by 3 days of continuous illumination under fluorescent light (62).

### Virulence assay

Conidia were harvested from SDC agar cultures and adjusted to a concentration of 8 × 10^4^ spores/mL in a 0.2% (wt:vol) gelatin solution. For pathogenicity assays, 2o-week-old seedlings of rice (*Oryza sativa* cv.CO39) were used, and 5 mL of conidial suspension of each treatment was sprayed onto the rice. Plants were incubated in a growth chamber at 28°C with 90% humidity and in the dark for the first 24 h, followed by a 12 h/12 h light/dark cycle. The disease severity was assessed at 7 days (63). Relative fungal growth in rice leaves was used to synthetically evaluate the disease severity. For the “relative fungal growth” assay, total DNA was extracted from 1.5 g disease leaves and tested by qRT-PCR with *M. oryzae* 28S ribosomal gene and *RUBQ1* (rice ubiquitin 2) primers (64).

### Yeast two-hybrid assay

Full-length cDNA *MoMKK1* was cloned into pGADT7 as a bait construct, and the cDNA of *MoATG9* or *MoATG6* gene was cloned into pGBKT7 as the prey construct. The resulting prey and bait constructs were first confirmed by sequencing analysis and then transformed in pairs into yeast strain AH109. Next, transformants grown on a synthetic dextrose medium lacking leucine and tryptophan (SD-Leu-Trp) for 3 days, and individual colonies were replicated to a synthetic medium lacking leucine, tryptophan, adenine, and histidine (SD-Leu-Trp-Ade-His) (65).

### Protein extraction and western blot analysis

The fusion construct was transferred into Guy11 and the Δ*Moatg9* mutant. Strains were cultured in liquid CM media for 36 h and then moved into nutrition starvation conditions (MM-N media) for 2 or 5 h. For the germinating process protein, we collected germinating conidia on an inductive surface at 5 h and froze in liquid nitrogen prepared for protein extraction (66).

The mycelia or germinating conidia were ground into a fine powder in liquid nitrogen and resuspended in 1 mL RIPA lysis buffer II (Sangon Biotech, C510006) with 2 mM PMSF (Beyotime Biotechnology, ST506-2) for total protein extraction. The cell lysates were placed on the ice for 30 min and shaken once every 10 min for protein extraction, followed by centrifugation at 13,000 *g* for 10 min at 4°C. We collected the supernatant lysates as total proteins. Samples were analyzed by 12% SDS-PAGE followed by western blotting with the GFP antibody (Abmart, 293967) or RFP antibody (Chromotek, 6g6-150) for protein analysis (66).

### Phosphorylation analysis through Phos-tag gel

The MoAtg9-GFP, MoAtg9^6A^-GFP, MoAtg9^5A^-GFP fusion construct was introduced into the Δ*Moatg9* mutant strain. The total protein extracted from mycelium was resolved on 8% SDS-PAGE prepared with 50 µM Phos-tag (NARD institute Limited company, 18D01) and 100 µM MnCl_2_. Gel electrophoresis was first performed with a constant voltage of 80 V for 8 h. Then, the gel was equilibrated in transfer buffer with 5 mM EDTA for 20 min two times and followed by transfer buffer without EDTA for another 20 min. Protein transfer from the Mn^2+^-phos-tag acrylamide gel to the PVDF membrane was still performed with 80 V for 48 h at 4°C, and then the membrane was analyzed by western blotting using the anti-GFP antibody (67).

### *In vitro* phosphorylation analysis

The GST-MoMkk1, His-MoAtg1, His-MoAtg9, His-MoAtg9^6A^, His-MoAtg9^5A^ were expressed in *Escherichia coli* BL21 cells. *In vitro*, the rapid and cost-effective fluorescence detection in tube (FDIT) method was used to analyze protein phosphorylation with the Pro-Q Diamond Phosphorylation Gel Stain (Thermo Fisher Scientific, P33301), a widely used phosphor-protein gel-staining fluorescence dye. For protein kinase reaction, 0.2 µg MoMkk1 (MoAtg1) was mixed with 2 µg MoAtg9 or MoAtg9^6A^ (MoAtg9^5A^), in a kinase reaction buffer (100 mM PBS, pH 7.5, 10 mM MgCl_2_, 1 mM ascorbic acid, with the appearance of 50 µM ATP) at room temperature (RT) for 60 min, followed by 10-fold of cold acetone was added to stop the reaction. Then, the protein was precipitated in a −20°C freezer for 4 h and centrifuged at 13,200 *g* for 1 h at 4°C. Phosphorylation protein was stained by 100 µL of Pro-Q Diamond Phosphorylation Gel Stain (Thermo Fisher Scientific, P33301) and kept in the dark at RT for 1 h. Then, the sample was added 10-fold of cold acetone and precipitated in a −20°C freezer for 4 h and centrifuged at 13,200 *g* for 1 h at 4°C again. The protein was washed with 0.5 mL cold acetone twice and dissolved in 200 µL of Mili-Q water after air-drying. The fluorescence signal was measured in a Cytation3 microplate reader (Biotek, Winooski, VT, USA) at 590 nm (excited at 530 nm) (68).

### Mass spectrometric analysis

To identify phosphorylation sites of targeted proteins, total proteins were extracted from Guy11/*MoATG9*, Δ*Momkk1*/*MoATG9,* and Δ*Moatg1*/*MoATG9* strains. Approximately 30 µL of anti-GFP beads (KT HEALTH, KTSM1301) was added into 1 mL protein samples. After incubation at 4 ˚C for 2 h, the beads were washed 3 times with 700 µL PBS, and proteins were eluted with 90 µL elution buffer (0.2 M glycine, pH 2.5). The eluent was immediately neutralized with 10 µL neutralization buffer (1.5 M Tris, pH 9.0). The eluted proteins were resolved on 12% SDS-PAGE gel to separate (69). The targeted protein bands were excised from the gel and subject to mass spectrometry analysis.

### Epifluorescence microscopy

*M. oryzae* cells (hyphae, conidia, or appressorium) expressing fluorescent protein-fused chimera were incubated under appropriate conditions. For appressoria, conidia were incubated on an inductive surface at 2 and 4 h. The constructs including RFP-MoApe1, RFP-MoAtg8, MoAtg9-GFP, MoAtg9^6A^-GFP, MoAtg9^6D^-GFP, and other phosphorylation mutation MoAtg9^5A^-GFP, MoAtg9^5D^-GFP were transformed into the Δ*Moatg9* mutant or the wild-type Guy11 strain. Epifluorescence microscopy was performed using a Zeiss LSM710, 63× oil microscope.

For time-lapse imaging experiments, strains were cultured in nutrition starvation conditions for 45 min and stained with the CellTracker Blue CMAC (LMAl Bio, LM-155) for 30 min.

### Filipin fluorescence staining assay

To test the sterol binding ability, MoAtg9-GFP, MoAtg9^6A^-GFP, MoAtg9^6D^-GFP, MoAtg9^5A^-GFP, MoAtg9^5D^-GFP, and GFP (negative control) were purified using anti-GFP beads and incubated with 10 µM ergosterols (Solarbio, SE8200) for 45 min and then stained with 50 µg/mL filipin (Cayman Chemical, 70440) for 4 h and observed by fluorescence microscopy with a UV filter. Before imaging, the beads were washed three times with PBS and then viewed under a UV filter set (50).

### Lipid scramblase assay

The unilamellar liposomes were prepared as described (39, 53, 54). Phosphatidylcholine and phosphatidylglycerol (Avanti Polar lipids, 850457, 840457) were mixed at a molar ratio of 9:1, dried using a rotary evaporator, and chloroform completely eliminated in a vacuum desiccator. Then, the lipid film was resuspended in buffer A (50 mM HEPES-NaOH, pH 7.4, 100 mM NaCl) and sonicated in a water bath for 10 min with a frequency of 40 kHz. Next, suspension was extruded 10 times through a 400 nm pore-size membrane and 4 times through a 200 nm pore-size membrane. Liposomes, NBD-PE (Avanti, 810153), and the purified protein were incubated for the incorporation of the fluorescently labeled lipid (53).

Fifty microliters of fluorescently labeled lipid was supplemented in 1,950 µL buffer A, and then, the sample was monitored using fluorescence at excitation and emission wavelengths of 470 nm and 530 nm, respectively. Forty microliters of the 1 M dithionite solution was added after 200 s followed by the addition of 0.1% Tween20 after 900 s (39).

### FRAP assay

FRAP experiments were performed on confocal microscopes (ZEISS LSM 980 with Airyscan2) to assess the autophagosome. Strains were cultured in nutrition starvation conditions for 45 min. Photobleaching was performed using 594 nm laser pulses (3 repeats, 20% intensity, dwell time 2.0 s) for the autophagosome.

For kinetic analysis, relative fluorescence intensity was recorded with time by setting the intensity before quenching as 1.0 and the other intensity after quenching as a ratio of t(s)/t0 (70).

## Data Availability

The genes from this study can be found in the GenBank database (https://www.ncbi.nlm.nih.gov/protein/) using the following accession numbers: MoAtg1 (MGG_06393), MoAtg9 (MGG_09559), MoAtg8 (MGG_01062), MoApe1 (MGG_07536), MoMkk1 (MGG_06482), MoAtg6 (MGG_03694).

## SUPPLEMENTAL MATERIAL

Fig. S1 mbio.03344-23-s0001.docx Yeast-two-hybrid (Y2H) analysis of the interaction between MoMkk1 and MoAtg9.

Fig. S2 mbio.03344-23-s0002.docx MoAtg9 phosphorylation is essential for the development of *M. oryzae*.

Fig. S3 mbio.03344-23-s0003.docx MoAtg9 phosphorylation is essential for the virulence of *M. oryzae*.

Fig. S4 mbio.03344-23-s0004.docx Subcellular localization of autophagosomes in conidia and appressorium.

Fig. S5 mbio.03344-23-s0005.docx Autophagosome entrance in vacuoles.

Fig. S6 mbio.03344-23-s0006.docx The different sites phosphorylated by MoMkk1 and MoAtg1 in the autophagy, development, and pathogenicity of *M. oryzae*.

ASM does not own the copyrights to Supplemental Material that may be linked to, or accessed through, an article. The authors have granted ASM a non-exclusive, world-wide license to publish the Supplemental Material files. Please contact the corresponding author directly for reuse.
