# Supplementary material for: MoMkk1 and MoAtg1 dichotomously regulating autophagy and pathogenicity through MoAtg9 phosphorylation in Magnaporthe oryzae
Source: mBio. 2024 Mar 19;15(4):e03344-23. doi: 10.1128/mbio.03344-23 (PMC11005334; doi:10.1128/mbio.03344-23)
Supplement: Fig. S1 — Yeast-two-hybrid (Y2H) analysis of the interaction between MoMkk1 and MoAtg9. [file mbio.03344-23-s0001.docx]

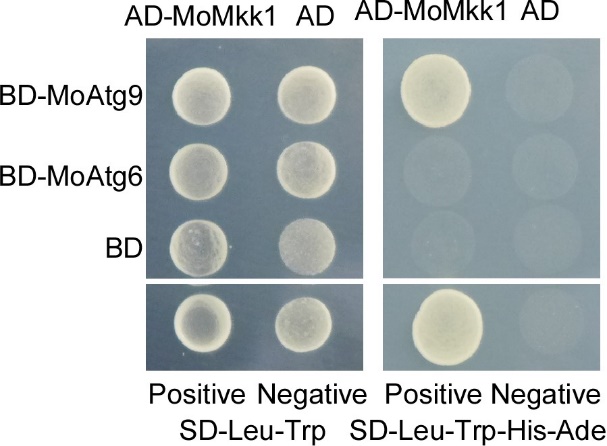


**Figure S1. Yeast-two-hybrid (Y2H) analysis of the interaction between MoMkk1 and MoAtg9.** MoAtg6 was used as a negative control that failed to interact with MoMkk1.
