# Supplementary material for: MoMkk1 and MoAtg1 dichotomously regulating autophagy and pathogenicity through MoAtg9 phosphorylation in Magnaporthe oryzae
Source: mBio. 2024 Mar 19;15(4):e03344-23. doi: 10.1128/mbio.03344-23 (PMC11005334; doi:10.1128/mbio.03344-23)
Supplement: Fig. S2 — MoAtg9 phosphorylation is essential for the development of M. oryzae. [file mbio.03344-23-s0002.docx]

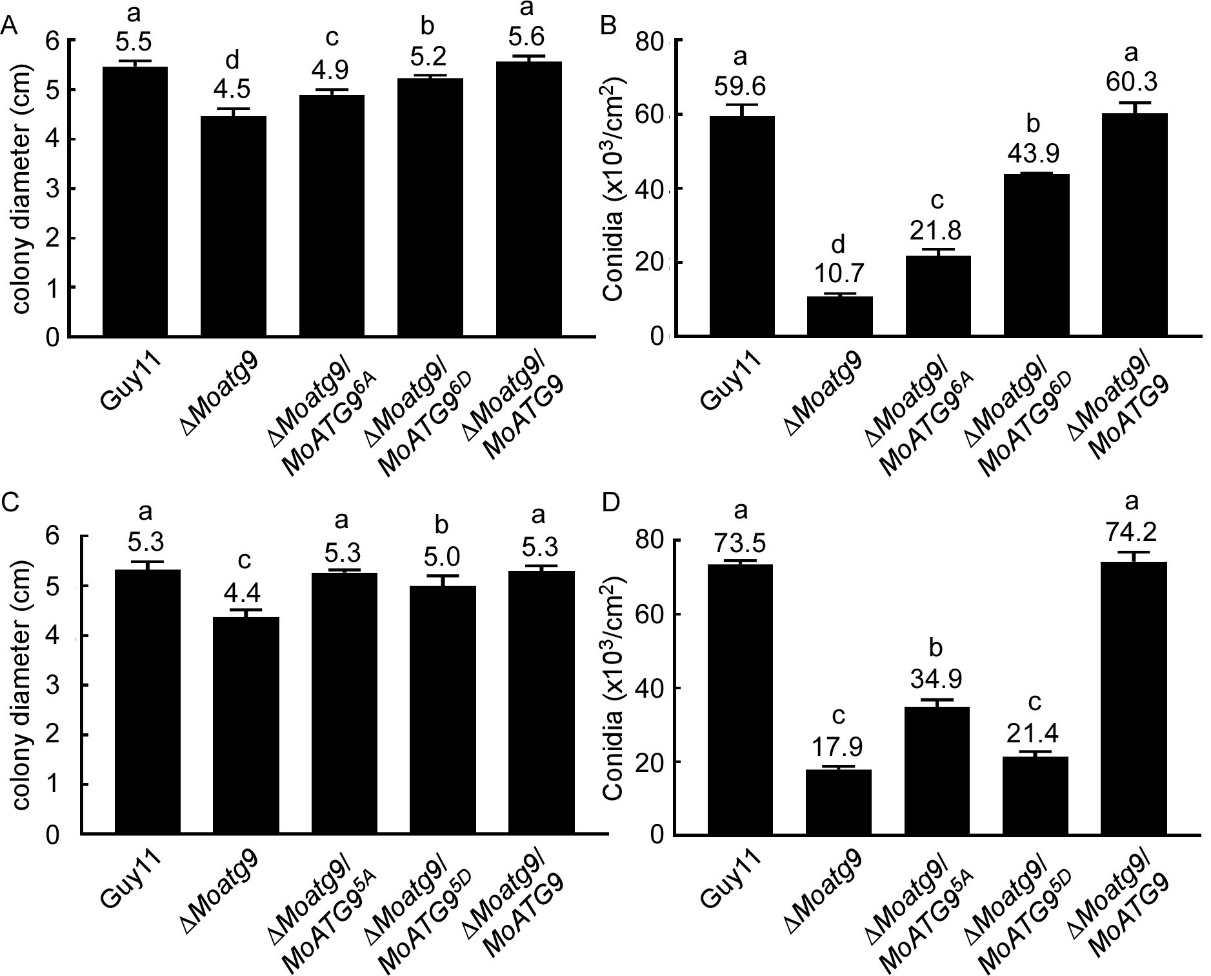


**Figure S2.** **MoAtg9 phosphorylation is essential for the development of *M. oryzae*.** (A) Statistical analysis of colony diameters from Guy11, Δ*Moatg9*, Δ*Moatg9*/*MoATG9^6A^*, Δ*Moatg9*/*MoATG9^6D^*, and Δ*Moatg9*/*MoATG9* strains on complete media (CM). Error bars represent SD, and different letters indicate statistically significant differences (Duncan’s new multiple range test, *p* < 0.05). (B) Statistical analysis of conidia production from Guy11, Δ*Moatg9*, Δ*Moatg9*/*MoATG9^6A^*, Δ*Moatg9*/*MoATG9^6D^*, and Δ*Moatg9*/*MoATG9* strains on SDC medium cultured at 28°C for 7 d in the dark followed by 3 d of continuous illumination under fluorescent light. Error bars represent SD, and different letters indicate statistically significant differences (*p* < 0.05). (C) Statistical analysis of colony diameters from Guy11, Δ*Moatg9*, Δ*Moatg9*/*MoATG9^5A^*, Δ*Moatg9*/*MoATG9^5D^*, and Δ*Moatg9*/*MoATG9* strains on CM. Different letters indicate statistically significant differences (*p* < 0.05). (D) Statistical analysis of conidia production from Guy11, Δ*Moatg9*, Δ*Moatg9*/*MoATG9^5A^*, Δ*Moatg9*/*MoATG9^5D^*, and Δ*Moatg9*/*MoATG9* strains. Different letters indicate statistically significant differences (*p* < 0.05).
