# Supplementary material for: MoMkk1 and MoAtg1 dichotomously regulating autophagy and pathogenicity through MoAtg9 phosphorylation in Magnaporthe oryzae
Source: mBio. 2024 Mar 19;15(4):e03344-23. doi: 10.1128/mbio.03344-23 (PMC11005334; doi:10.1128/mbio.03344-23)
Supplement: Fig. S3 — MoAtg9 phosphorylation is essential for the virulence of M. oryzae. [file mbio.03344-23-s0003.docx]

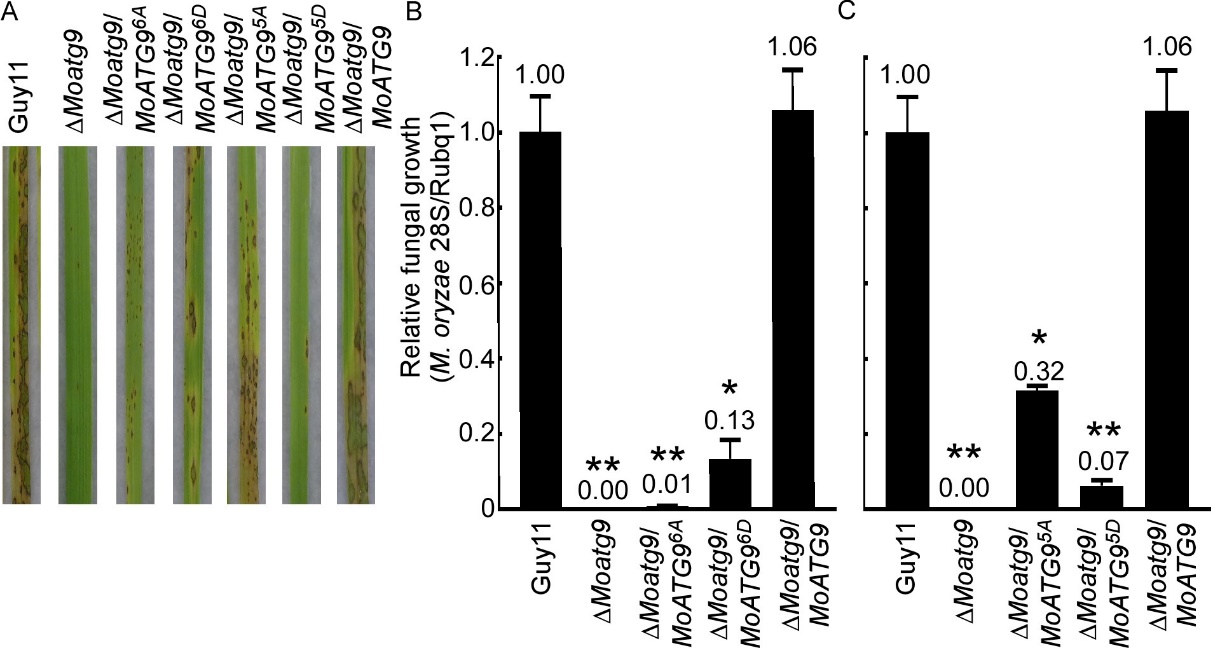


**Figure S3.** **MoAtg9 phosphorylation is essential for the virulence of *M. oryzae*.** (A) Five milliliters of conidial suspension (8×10^4^ spores/mL) of each strain were used for spraying and photographed 7 d. (B, C) The severity of blast disease was evaluated by quantifying *M. oryzae* genomic 28S rDNA relative to rice genomic *Rubq1* DNA (7 d). Mean values of three determinations with standard deviations are shown. Error bars represent SD, and asterisks indicate a significant difference from the wild-type Guy11 (*p* < 0.01).
