# Supplementary material for: MoMkk1 and MoAtg1 dichotomously regulating autophagy and pathogenicity through MoAtg9 phosphorylation in Magnaporthe oryzae
Source: mBio. 2024 Mar 19;15(4):e03344-23. doi: 10.1128/mbio.03344-23 (PMC11005334; doi:10.1128/mbio.03344-23)
Supplement: Fig. S4 — Subcellular localization of autophagosomes in conidia and appressorium. [file mbio.03344-23-s0004.docx]

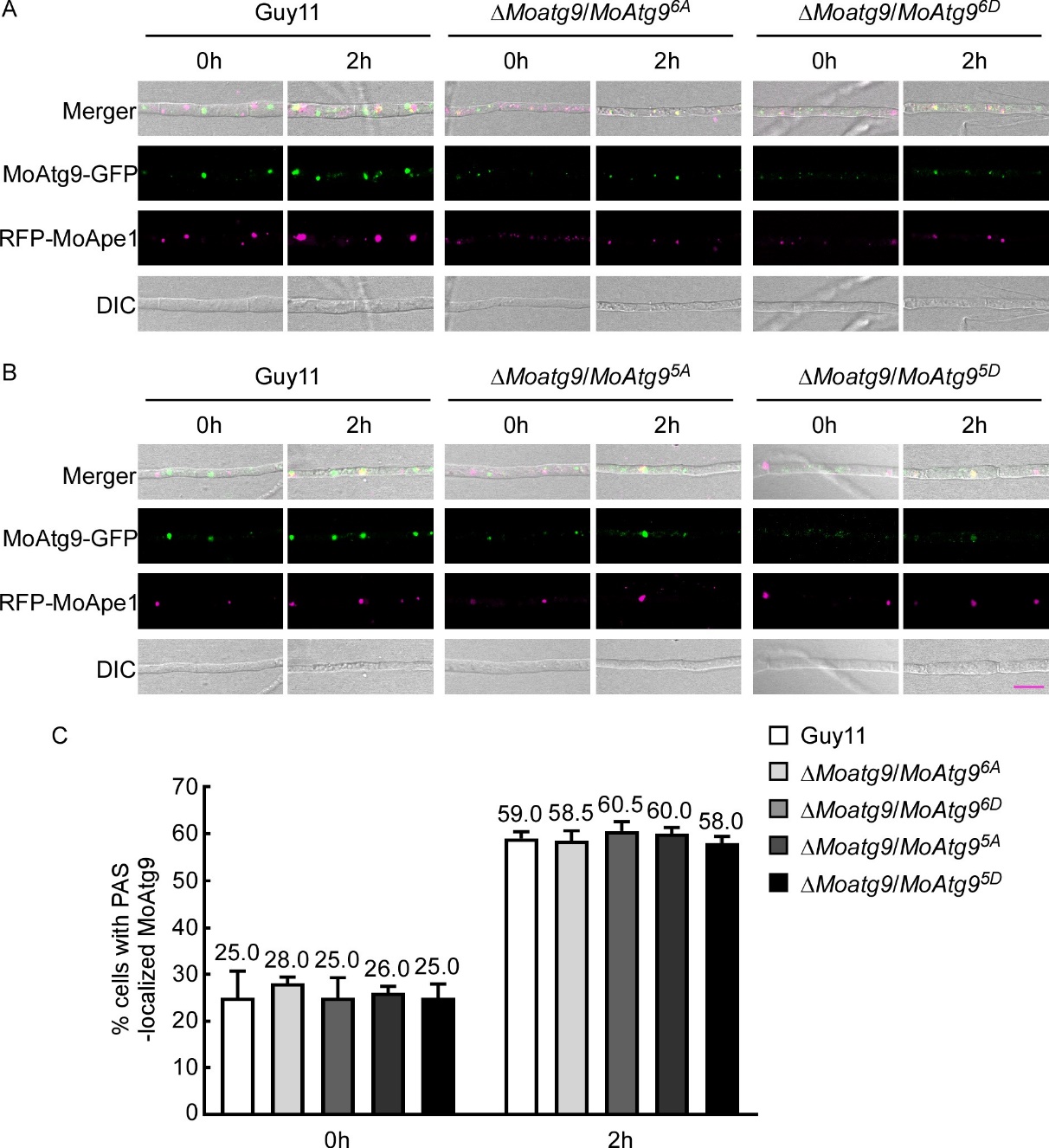


**Figure S4.** **Subcellular localization of MoAtg9-GFP in nitrogen starvation for 0 and 2 h.** (A) RFP-MoApe1 marked PAS can be used as an autophagic marker protein. Light microscopic examination of distributions of MoAtg9-GFP and RFP-MoApe1 in Guy11, Δ*Moatg9*/*MoATG9^6A^*, and Δ*Moatg9*/*MoATG9^6D^*. (B) Light microscopic examination of distributions of MoAtg9-GFP and RFP-MoApe1 in Guy11, Δ*Moatg9*/*MoATG9^5A^*, and Δ*Moatg9*/*MoATG9^5D^*. Scale bar, 10µm. (C) The localization ratio in PAS of Guy11, Δ*Moatg9/MoATG9^6A^*, Δ*Moatg9/MoATG9^6D^*, Δ*Moatg9/MoATG9^5A^*, and Δ*Moatg9/MoATG9^5D^* under nutrient-rich and poor conditions. Error bars represent SD.
