# Supplementary material for: MoMkk1 and MoAtg1 dichotomously regulating autophagy and pathogenicity through MoAtg9 phosphorylation in Magnaporthe oryzae
Source: mBio. 2024 Mar 19;15(4):e03344-23. doi: 10.1128/mbio.03344-23 (PMC11005334; doi:10.1128/mbio.03344-23)
Supplement: Fig. S5 — Autophagosome entrance in vacuoles. [file mbio.03344-23-s0005.docx]

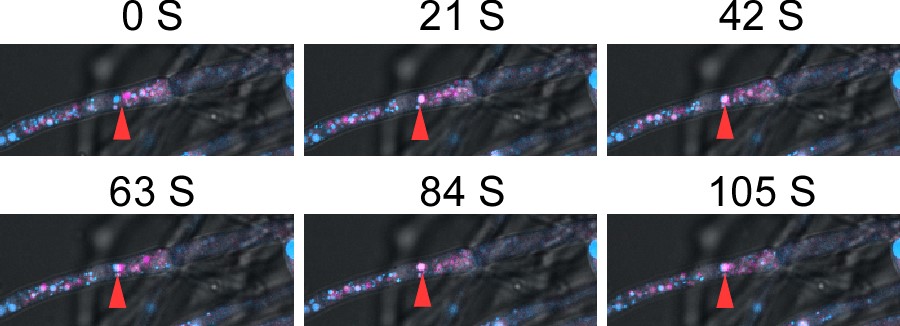


**Figure S5. Autophagosome entrance in vacuoles.** Vacuoles were stained with CMAC. RFP-MoAtg8 translocation from the cytoplasm to the vacuole during MM-N (45 min).
