# Supplementary material for: MoMkk1 and MoAtg1 dichotomously regulating autophagy and pathogenicity through MoAtg9 phosphorylation in Magnaporthe oryzae
Source: mBio. 2024 Mar 19;15(4):e03344-23. doi: 10.1128/mbio.03344-23 (PMC11005334; doi:10.1128/mbio.03344-23)
Supplement: Fig. S6 — The different sites phosphorylated by MoMkk1 and MoAtg1 in the autophagy, development, and pathogenicity of M. oryzae. [file mbio.03344-23-s0006.docx]

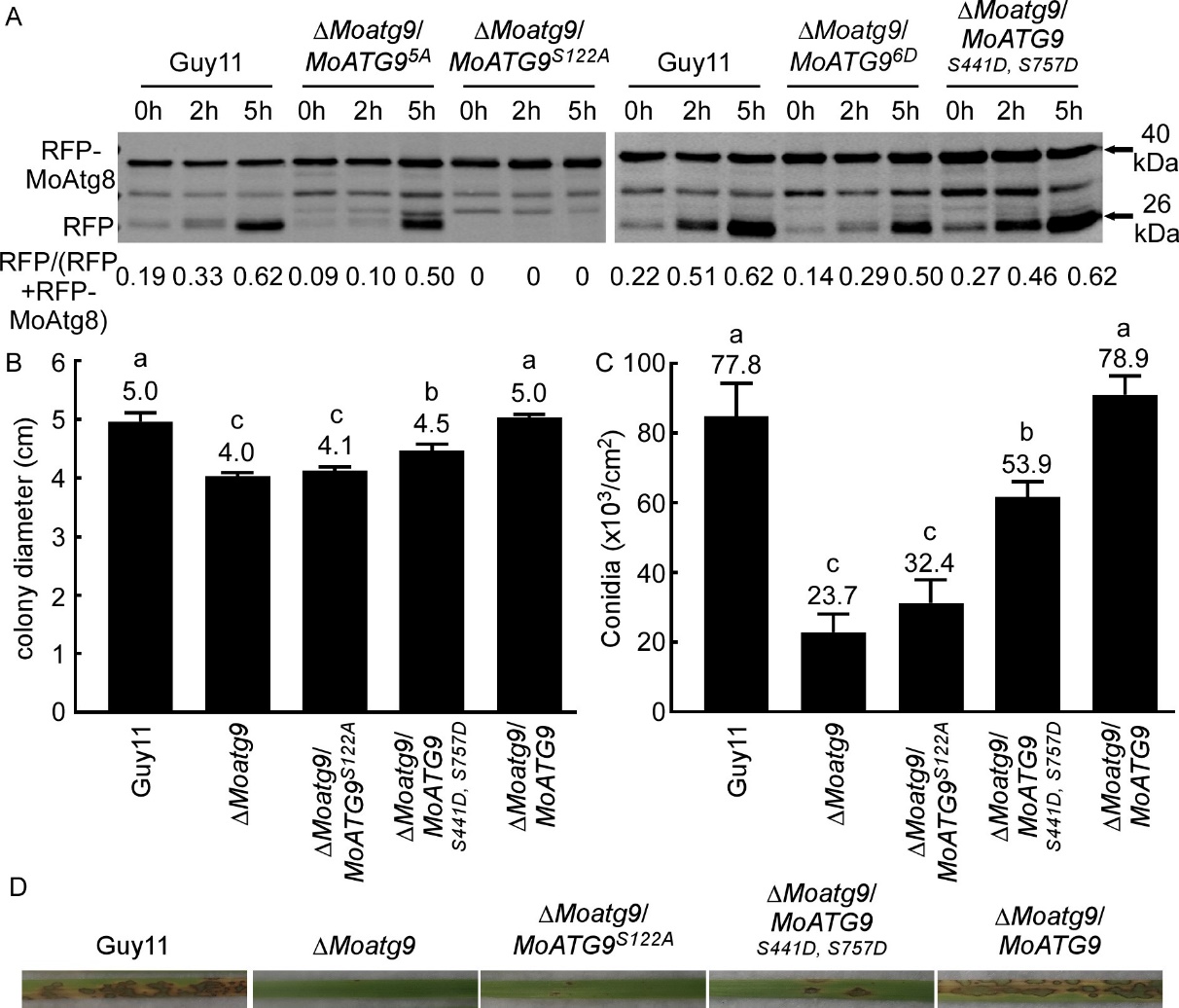


**Figure S6. The different sites phosphorylated by MoMkk1 and MoAtg1 in the autophagy, development, and pathogenicity of *M. oryzae*.** (A) Guy11, Δ*Moatg9*, Δ*Moatg9/MoATG9^S122A^*, and Δ*Moatg9/MoATG9^S441D, S757D^* strains transformed with RFP-MoAtg8 were cultured in MM-N for 0, 2, and 5 h. The extent of autophagy was estimated by calculating the amount of free RFP compared with the total amount of intact RFP-MoAtg8 and free RFP (the numbers underneath the blot). (B) Statistical analysis of colony diameters from Guy11, Δ*Moatg9*, Δ*Moatg9/MoATG9^S122A^*, Δ*Moatg9/MoATG9^S441D, S757D^*, and Δ*Moatg9/MoATG9* strains on CM. Different letters indicate statistically significant differences (*p* < 0.05). (C) Statistical analysis of conidia production from Guy11, Δ*Moatg9*, Δ*Moatg9/MoATG9^S122A^*, Δ*Moatg9/MoATG9^S441D, S757D^*, and Δ*Moatg9/MoATG9* strains. Different letters indicate statistically significant differences (*p* < 0.05). (D) Five milliliters of conidial suspension (8×10^4^ spores/mL) of each strain were used for inoculation. The lesions were photographed 7 d.
